# The Impact of Pain Assessment on Critically Ill Patients' Outcomes: A Systematic Review

**DOI:** 10.1155/2015/503830

**Published:** 2015-10-19

**Authors:** Evanthia Georgiou, Maria Hadjibalassi, Ekaterini Lambrinou, Panayiota Andreou, Elizabeth D. E. Papathanassoglou

**Affiliations:** ^1^Education Sector, Nursing Services, Ministry of Health, 1 Prodromou & Chilonos Street 17, 1448 Nicosia, Cyprus; ^2^Cyprus University of Technology Department of Nursing, 15 Vragadinou Street, 3041 Limassol, Cyprus

## Abstract

In critically ill patients, pain is a major problem. Efficient pain management depends on a systematic, comprehensive assessment of pain. We aimed to review and synthesize current evidence on the impact of a systematic approach to pain assessment on critically ill patients' outcomes. A systematic review of published studies (CINAHL, PUBMED, SCOPUS, EMBASE, and COCHRANE databases) with predetermined eligibility criteria was undertaken. Methodological quality was assessed by the EPHPP quality assessment tool. A total of 10 eligible studies were identified. Due to big heterogeneity, quantitative synthesis was not feasible. Most studies indicated the frequency, duration of pain assessment, and types of pain assessment tools. Methodological quality assessment yielded “strong” ratings for 5/10 and “weak” ratings for 3/10 studies. Implementation of systematic approaches to pain assessment appears to associate with more frequent documented reports of pain and more efficient decisions for pain management. There was evidence of favorable effects on pain intensity, duration of mechanical ventilation, length of ICU stay, mortality, adverse events, and complications. This systematic review demonstrates a link between systematic pain assessment and outcome in critical illness. However, the current level of evidence is insufficient to draw firm conclusions. More high quality randomized clinical studies are needed.

## 1. Introduction

Despite several decades of research, pain is still a significant problem for critically ill patients throughout their stay in the intensive care unit (ICU). Inaccurate pain assessment and the resulting inadequate treatment of pain in critically ill adults can have significant physiological and psychological consequences. Underdiagnosed pain has been linked to a number of adverse outcomes including increased infection rate, prolonged mechanical ventilation, hemodynamic derangements, delirium, and compromised immunity [1–3]. Appropriate pain management depends on the systematic and comprehensive assessment of pain to guide decision-making regarding titration of analgesia and administration of “as needed” medications [4, 5]. However, the extent to which systematic pain assessment may in itself have an effect on critically ill patients' outcomes remains unclear. Despite the existence of recommendations and guidelines on the subject of pain assessment and management as well as the existence of validated tools to measure pain in verbal and nonverbal patients, their implementation and use in routine clinical practice appear to be scarce and inconsistent [6]. Moreover, although most ICUs have protocols for pharmacological pain management in place, or even* pro re nata* (*prn*) medical orders, the means to assess the presence and intensity of pain in critically ill individuals have been inconsistent, therefore limiting the benefit of analgesia protocols [7].

## 2. Aim

This study focuses on pain assessment as an independent intervention, and it aims to review current evidence onhealth related outcomes of the systematic use of pain assessment tools in the ICU. Evaluation of the efficacy of different pain management protocols is beyond the scope of this review, which targets to explore the impact of systematic monitoring of pain indicators in the ICU. The study was driven by the following research question formulated according to the PICO methodology (Patient, Intervention, Comparison, and Outcome) [8].

“In critically Ill adults does implementation of a systematic assessment of pain, compared to no systematic assessment, improve patients' outcomes with regard to (a) level of pain and agitation, (b) appropriate use of analgesics and sedatives, (c) length of ICU stay, (d) complications and adverse events, (e) duration of mechanical ventilation and days off ventilator, (f) patient satisfaction, and (g) mortality?.”

## 3. Background

Although pain is reported as a predominant stressor that can activate many pathophysiological mechanisms in critically ill patients, assessment rates of pain in ICU remain low [18]. This is mainly due to critically ill patients' inability to communicate their pain, due to either sedation, or cognitive impairment, paralysis, or mechanical ventilation. The multicenter patient-based DOLOREA study described current practices in analgesia and sedation use for 1,381 mechanically ventilated patients during their first week in the ICU. It was found that only 42% of patients received pain assessments on day 2 (D2) in ICUs, although 90% of patients were concomitantly given opioids [19]. The positive association between the ability to assess and document patients' pain and the sufficient management of pain has been previously documented [20, 21]. Inaccurate pain assessment and the ensuing inadequate treatment of pain in critically ill adults can affect all bodily systems with a plethora of physiologic and psychological consequences [22, 23] involving short- and long-term clinical outcomes of critically ill patients [24].

Inconsistent use of standardized tools has been recognized as a leading factor in inadequate pain control. Gélinas and coworkers [25] found that of 183 documented pain episodes in intubated patients, use of a pain scale was mentioned in only 1.6% of cases. Although assessment of pain behaviors was common (73% of episodes), these assessments were observed and documented without the use of a valid and reliable tool. In another investigation of 1,360 mechanically ventilated critically ill patients, Payen and coworkers [19] found that pain was not assessed in 53% of patients who were receiving analgesia, and when pain was assessed, specific pain tools were used only 28% of the time. In addition, in a recent multicentre prospective observational study more than 50% of the included ICUs reported the availability of pain assessment tools and protocols for the management of pain; however their application was rare (pain scale 19.1% and protocol 25.0%) [26].

In the “clinical practice guidelines for the management of pain, agitation and delirium in the critically ill adult” published by the Society of Critical Care Medicine (SCCM) it is recommended that pain is routinely monitored in all adult ICU patients [27]. Patient self-report through use of the numeric rating scale (NRS) ranging from 0 to 10 is widely recognized as the best pain assessment tool. However, in case of noncommunicative patients, use of a valid and reliable behavioral pain tool, like the behavioral pain scale (BPS) and the critical-care pain observation tool (CPOT) [27], is suggested. It is widely accepted that the use of reliable behavioral pain assessment tools can assist health care providers in the early identification of pain in critically ill patients and subsequently in the prompt and efficient management of pain [5, 28].

## 4. Materials and Methods

### 4.1. Search Strategy

A focused literature search was undertaken with consultation with a professional librarian, from December 2013 to March 2014. CINAHL, PUBMED, SCOPUS, EMBASE, and COHRANE databases were searched using the terms “pain assessment” OR “pain evaluation” AND “critical care unit” OR “intensive care unit” AND “pain assessment tools” OR “pain assessment scales” AND “mechanical ventilation” OR “length of stay” OR “level of pain” OR “agitation” OR “hypnotics and sedatives” OR “analgesic drugs” OR “complications and adverse events” OR “mortality” OR “patient satisfaction.” References of identified studies were also checked for relevancy. Hand-searches of relevant journals and search of gray literature (Open Archives.gr, Proquest Dessertations and Thesis) were also performed.

**Figure 1:**
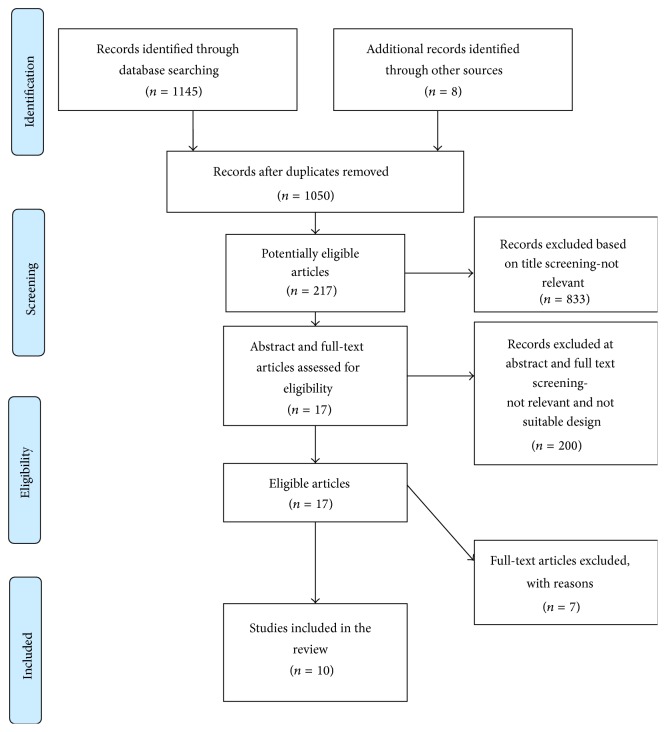
Flowchart of the literature search and article selection process.

After searching all sources, 1,153 articles were accumulated and exported into reference manager software, refworks (ProQuest for Word version 4.3). This permitted easy removal of duplicates (*n* = 103). Study titles were then screened (electronically using the reference manager) independently by two reviewers who selected studies appropriate for inclusion in the review (*n* = 217). During the next phase abstracts and full text of the retrieved articles were read and compared by the reviewers against to the inclusion and exclusion criteria. During this phase, 200 articles were excluded. Finally, 17 articles were considered for inclusion in the review. After consultation with a third reviewer and consensus, 7 more were excluded with reasons either of not appropriate design (3 studies) or not relevant outcome measures (4 studies). This effort resulted in 10 eligible articles (Figure 1). Subsequently, data were extracted, summarized, and analyzed. Prisma guidelines were employed to guide analysis and reporting of results.

### 4.2. Selection Criteria

The decision to include studies was based on predetermined criteria. Inclusion criteria comprised: (a) a study population consisting of adult critically ill patients within the ICU, irrespective of their ability to communicate and whether they were on mechanical ventilation support or not, (b) implementation of one or more pain assessment tools as single intervention, and (c) reporting the effect of pain assessment practice on patient outcomes including one or more of the following: level of pain, duration of mechanical ventilation, days off ventilator, length of ICU stay, patient satisfaction, complications and adverse events, appropriate use of analgesics and sedatives, and mortality.

No time or language limitations were set. Studies employing concurrent interventions including a formal protocol prescribing interventions for the management of pain, either pharmacological and/or nonpharmacological, were excluded, in order to avoid the confounding effect of additional interventions. Studies investigating children or neonates were excluded.

### 4.3. Quality Assessment of Selected Studies

The Quality Assessment Tool for Quantitative Studies (QATQS), Effective Public Health Practice Project Quality assessment tool (EPHPP-2008) [29], was employed to score identified studies in order to enhance the consistency of results [30]. QATQS was chosen because it includes questions related to nonrandomized and observational designs such as quasiexperimental and before-and-after studies. It has been reported as a valid tool for assessing the quality of a study and for making comparisons between studies, while addressing major threats to validity [31–33]. QATQS employs five criteria which evaluate the likelihood of selection bias, quality of study design, presence of confounding variables, validity and reliability of the method of data collection, and the number of withdrawals and dropouts [29]. The overall study quality is considered to be strong if none of the quality domains is rated as weak, moderate if one domain is rated as weak, and weak if two or more domains are rated as weak. The methodological quality of the identified articles was independently assessed by two reviewers. Each reviewer assessed and rated all the studies independently based on the above assessment tool. Reviewers then went through each criterion and compared the scores of each study noting down any differences. The level of interrater agreement was assessed by the kappa coefficient. A third reviewer was appointed from the beginning of the study, in order to resolve potential disagreements between assessors using the consensus method.

## 5. Results

### 5.1. Quality Assessment

An almost perfect interrater agreement was observed, with kappa coefficient for each of the six component ratings ranging from 0.79 to 0.87 (*P* < 0.0001) for all studies. Methodological quality assessment on the basis of the EPHPP quality assessment tool yielded “strong” ratings for the 5/10 included studies, “moderate” ratings for 2/10, and “weak” ratings for 3/10 (Table 2). Owing to the preexperimental, pretest-posttest nature of designs, several threats to validity are potentially present, involving selection bias and interactions with history, testing, instrumentation, and regression to the mean.

### 5.2. Characteristics of Participants

The identified studies were published between 1995 and 2013 and they were conducted in different countries (Canada: 4 studies; Europe: 2 studies; USA: 2 studies; Australia: 1 study). With regard to study design, 9/10 studies employed a preexperimental pretest-posttest approach, and one study employed a two-group comparative approach (Table 1). No studies employing randomized controlled trial designs (RCT) were identified. The number of participants included ranged from 30 [11] to 1144 [6]. All studies involved adult ICU patients from either surgical [10], mixed [6, 9, 12, 14, 15], cardiothoracic [16, 17], trauma [9, 11, 13], or neurosurgical ICUs [13]. Only 6/10 studies reported the use of a scoring system for the assessment of incidence of organ dysfunction/failure and prediction of outcome of the critically ill patients, as a baseline measurement (Table 1). Two studies included patients who were not intubated and were able to communicate [16, 17], and 5 studies included noncommunicative patients [6, 9, 11, 12, 14], either on mechanical ventilation [11, 14] or not [13]. Topolovec-Vranic et al. [13] assessed a mixed population of communicative and not communicative patients and two studies included consecutive ICU admissions on the basis of predetermined inclusion criteria [10, 15].

### 5.3. Characteristics of Studies and Limitations

The characteristics of the intervention and outcome measures varied; a detailed account is presented in Table 1. Interventions generally included guidance on frequency and duration of pain assessment and the method and choice of pain assessment tools.

Rose et al. [9] used a before and after design to evaluate the effectiveness of the introduction of C-POT in two ICUs, on the frequency of documentation of pain assessment and administration of analgesics and sedatives in critically ill patients unable to self-report pain. Data were recorded for a maximum of 72 hours before and after implementation of the tool in both units. The authors recognized several confounding factors that might have influenced their results (i.e., staff turnover, differences in patients characteristics, etc.) and potential performance and ascertainment bias.

Radtke et al. [10] conducted a nonrandomized study with before (for two months) and after measurements (for two months) and one-year follow-up to compare the effectiveness of two training strategies (training according to the local standard versus modified extended training) on the implementation rate of the numeric rating scale (NRS), BPS for pain assessment, Richmond Agitation sedation Scale (RASS), and the delirium detection score (DDS). Possible sources of bias were reported, including unequal and heterogeneous groups, selection bias and several confounding factors that may have had an impact on the results.

Arbour et al. [11] performed a pilot before-and-after study to explore the impact of the implementation of the CPOT on pain management among mechanically ventilated trauma intensive care unit patients. Data were collected from medical files 1 year before the implementation of the CPOT and 6 months after implementation. The exploratory nature of the study, along with the small heterogeneous sample and sensitization to measurement, was acknowledged as limitations by the authors.

Gélinas et al. [12] examined ICU nurses' reports of pain assessments, reassessments and of behaviors indicative of the presence of pain, as well as administration of analgesics/sedatives, and the effectiveness of pharmacological interventions pre- and postimplementation of the CPOT. A before and after study design was used, incorporating three phases: a three-month preimplementation phase including the review of 30 medical files, a three-month implementation phase during which nurses were trained in the use of CPOT, and a postimplementation phase. The retrospective review of medical files, inconsistencies of implementation differences in pretest/posttest tools and institutional changes during the study were acknowledged as limitations.

In the study by Topolovec-Vranic et al. [13] the Nonverbal Pain Scale (NVPS) was implemented and effects were explored through a pre-postdesign. Preimplementation data were gathered during a 4-week period. The NVPS documentation tool covered a 48-hour period. Nurses were instructed to assess and document patients' pain scores every 4 hours, as well as before and after a procedure (e.g., suctioning, turning), and to consider pain management options if the pain score was greater than 4. The choice of NVPS, due to its limited content validity and reliability as a pain measure for nonverbal patients [34], and also the potential selection bias for the patient satisfaction survey were reported as limitations.

Payen's et al. [6] study was a part of a multicenter prospective cohort study of mechanically ventilated patients who received analgesia on day 2 of their stay in the ICU. Propensity-adjusted score analysis was employed to compare the duration of ventilator support and duration of ICU stay between 513 patients who were assessed for pain and 631 patients who were not assessed for pain. A variety of pain assessment tools were used (BPS, VAS, verbal descriptor scale, NRS, and Harris scale). Limitations include nonadjustment for confounding factors and failure to account for interactions between sedatives and opioids.

Williams et al. [14] used a before-and-after design to evaluate results of implementation of the Behavioral Pain Scale and the Richmond Agitation-Sedation Scale for patients receiving mechanical ventilation. Data were collected for 6 months before and 6 months after training and introduction of the scales. Selection bias and failure to control for confounding factors were acknowledged as limitations.

Chanques and coworkers [15] used a pre-postintervention design and implemented use of BPS (for noncommunicative patients) in combination with NRS (for communicative patients) to evaluate pain. The preimplementation data collection was 21 weeks during which BPS, NRS, and RASS were measured twice daily, at rest, by independent observers. This was followed by a period of 4 weeks of training and a subsequent 29-week intervention phase, during which nurses assessed pain and agitation levels and notified physicians if BPS > 5 or NRS > 3 RASS > 1. Several limitations are acknowledged including unequal rates of measurements at the pre- and postimplementation phases and the fact that the incidence and intensity of pain and agitation events were observed only at rest.

Voigt et al. [16] through a pre-postintervention design examined the impact of nurses' use of a standardized pain flow sheet to document pain and pharmacologic management on the basis of NRS ratings as reported by patients. Additionally, within 24 hrs after transfer to the step-down unit, patients were interviewed regarding pain intensity experienced in the surgical heart unit and at the time of questioning. Lack of randomization and uncontrolled confounding factors was reported as limitations.

Meehan et al. [17] reviewed retrospectively and prospectively the charts of adult cardiac-surgical patients to examine nursing practice regarding analgesia and to compare patients' outcomes. The subjects completed the visual analogue scale (VAS) twice daily, as a measure of pain intensity and the Pain Relief Satisfaction Questionnaire on the day after transfer from the cardiothoracic ITU. Failure to account for a number of confounders and low generalizability to other ICU populations was acknowledged.

### 5.4. Main Outcomes

#### 5.4.1. Compliance and Documentation of Pain Assessments

Eight studies reported the impact of the pain assessment protocol on the documentation and compliance with systematic pain assessment practice. Documented reports of pain assessments and reassessments as well as reports of the presence of pain appeared to be more frequent after the implementation of pain assessment tools [9–13, 15, 16]. Further, in the study by Payen et al. [6], patients with regular pain assessments were more likely to be assessed for sedation and for procedural pain as well. Only two studies did not report on this outcome [14, 17].

Only in two studies, one involving trauma and neurosurgical [13] and the other mostly medical patients, measures of feasibility of measurements were assessed. Topolovec-Vranic et al. [13] noted that 78% of nurses reported that NVPS was easy to use and 80% were satisfied by its use, and Gélinas et al. [12] reported high interrated agreement after only a short training.

#### 5.4.2. Impact of Pain Assessment on Medications Used

Pain assessment appeared to influence the administration of medications in eight studies [6, 9, 11, 12, 14–17], with the majority of studies reporting better pain management and more efficient use of analgesics and/or sedatives. Only one study reported no difference in the type of opioid analgesics administered or the amount of medication (morphine equivalents) after implementation of the pain assessment tool [13].

In the study by Rose et al. [9], implementation of the pain assessment tool had different effects on opioid and benzodiazepine administration in the two participating ICUs. Specifically, in the cardiovascular ICU, a small but significant decrease in use of opioid analgesics and a large decrease in the administration of benzodiazepines occurred, while in the mixed ICU use of opioid analgesics increased dramatically, and benzodiazepine usage was unchanged. Similarly, in the study by Arbour et al. [11] analgesics were administered less often and morphine equianalgesic dosages were lower in the postimplementation compared to the preimplementation group. In the same study, while no statistically significant difference was found, patients of the preimplementation group received sedative agents twice as often. In another study, fewer analgesic and sedative agents were administered during the postimplementation phase [12]. In contrast, Williams et al. [14] reported more prolonged use of sedatives after implementation of the pain assessment protocol.

Chanques and coworkers [15] report significant changes involving both increase and decrease in doses of analgesic and psychoactive drugs, but no significant differences in continuous infusion of sedatives in the intervention group. Only the dose of morphine administered as continuous sedation showed a trend to be higher in the intervention group.

In the study by Meehan et al. [17], the preimplementation group received significantly more analgesia (morphine equivalents) through day 3, with the greatest difference being on day 1, while the postimplementation group received more analgesia later in their stay. Similarly, Voigt et al. [16] in a sample of cardiac surgery patients reported more pain medication received on day 1 at the postimplementation group. Payen et al. [6] reported that more patients with pain assessments were treated with nonopioids, compared to those without pain assessments and that they also received fewer hypnotics and lower daily doses of midazolam. Finally, two studies reported that patients who were assessed for their pain were more likely to have dedicated pain treatment during procedural pain events [6, 17].

#### 5.4.3. Impact of Pain Assessment on Level of Pain

Three studies examined the effect of systematic pain assessment on either intensity or incidence of pain experienced by critically ill patients, and they reported overall favorable outcomes.

Chanques and coworkers [15] reported that the incidence of pain, as well as the rate of severe pain events (NRS > 6), decreased significantly during the intervention phase. However, BPS pain scores and the rate of severe pain events based on the BPS (BPS > 7) were not significantly different compared to the preimplementation phase. Similarly, in the study by Voigt et al. [16] the distribution of pain intensity scores in the postintervention group shifted towards lower values, relative to the distribution of those in the preintervention group. This difference was statistically significant for the average and least amount of pain experienced during ICU stay and the pain experienced at the moment of questioning [16]. No pre-postimplementation comparisons were made in the study by Williams et al. [14]. Topolovec-Vranic et al. [13] reported decreased retrospective patient pain ratings after the implementation of the NVPS and a trend toward a decrease in the time required to receive pain medication. Patients' ratings of their “pain right now” on a scale from 0 to 10 did not differ from before to after implementation of the NVPS [13].

#### 5.4.4. Impact of Pain Assessment on Duration of Mechanical Ventilation

Eight studies examined the effect of systematic pain assessment on duration of mechanical ventilation, of which 2 studies reported significant decreases [6, 15]. Payen et al. [6] report increased odds for weaning from the ventilator (OR: 1.40) and decreases risk of ventilator- associated pneumonia (VAP) (OR: 0.75) in the group of patients routinely assessed for pain. Likewise, Chanques et al. [15] report significantly decreased risk of VAP. In the study by Arbour et al. [11], although no statistical difference was found in the duration of mechanical ventilation between the 2 groups, there was a clear trend for decreased duration of mechanical ventilation by approximately 3 days and almost half of patients in the preimplementation group (*n* = 7) were ventilated for a period of more than 96 hours as opposed to only 4 patients of the postimplementation group. However, interventions involving pain assessment practices were not associated with a significant reduction in duration of mechanical ventilation in 5 studies [9, 10, 14, 16, 17].

#### 5.4.5. Impact of Pain Assessment on Occurrence of Adverse Events and Complications

Four studies addressed the impact of pain assessment on the occurrence of adverse events and complications. Arbour et al. [11] reported that patients in the postimplementation group showed a significantly lower number of complications, compared to the preimplementation group, although they did not make specific mention to the type of complications. Similarly, Chanques and coworkers [15] observed a marked decrease in the overall nosocomial infections rate in the intervention compared to the control group, as well as significant decreases with regard to VAP, central catheter-related infections, urinary tract infections, and bacteremia. Likewise, Payen et al. [6] report significantly decreased rates of VAP, but no statistical significant differences with regard to thromboembolic events, gastroduodenal hemorrhage, and central venous catheter colonization. Nonetheless, in the study of Williams et al. [14] adverse events were equally distributed between the 2 groups except for the incidence of unplanned extubation which was less, but not statistically significant so, in the postimplementation group.

#### 5.4.6. Impact of Pain Assessment on Patient Satisfaction

Only two studies assessed the impact of pain assessment on patient satisfaction. In the study by Meehan et al. [17] almost all of the participants prospectively expressed their satisfaction with pain management in the cardiothoracic ICU. Topolovec-Vranic et al. [13] stated that both before and after implementation of systematic pain assessment, a large proportion of patients reported that their physician or nurse explained the importance of pain treatment to them, were satisfied with the way their nurses and physicians responded to their reports of pain and expressed their belief that the staff in the ICU did everything they could to help control their pain. However, after implementation of the NVPS, fewer patients reported a delay more than 5 minutes to receive pain medication when they requested it in the ICU.

#### 5.4.7. Impact of Pain Assessment on ICU Length of Stay (LOS)

Assessment of pain had no significant influence on ICU length of stay in 4 studies [10, 14–16]. However, in the study of Payen et al. [6] patients who were assessed for pain had a shorter duration of ICU stay, by 5 days on average (adjusted OR: 1.43). A trend for decreased ICU LOS was observed in two studies [9, 11]. Similarly, Meehan et al. [17] report a bigger percentage of patients (76%) in the prospective postimplementation group that were transferred on day 2 or sooner from the ICU compared with the retrospective preimplementation group (54%); this difference was statistically significant.

#### 5.4.8. Impact of Pain Assessment on Mortality

Only three studies investigated the impact of pain assessment on mortality. In one study [10], the implementation of pain monitoring and use of pain assessment tools were associated with a decrease in mortality (OR: 0.36), whereas in the other two studies there was no significant difference in mortality between the two groups of patients (OR: 0.8–0.91) [6, 15].

## 6. Discussion

This review identified studies exploring the impact of systematic pain assessment on critically ill patients' outcomes. Due to heterogeneity in study design, patient populations, interventions, setting, and time of study, quantitative synthesis was not possible. Several methodological limitations, including preexperimental design approaches, limited control of confounders and small sample sizes burden this body of evidence. However, data seem to indicate an association between systematic pain assessment and improved patients' outcomes. Despite the perceived importance of pain for both physiological and psychological outcomes in critical illness [35], the lack of studies assessing the impact of pain monitoring is astounding. Correspondingly, it is worth-noting that the results of this systematic review cannot be compared with other reviews as there are no systematic reviews or meta-analyses focusing on the impact of systematic pain assessment approaches on critically ill patients' outcomes. Only one relevant narrative review was identified, which examined the importance of pain assessment and its potential impact, as part of reviewing different strategies for the improvement of patients' outcomes [18].

The findings of this review support the notion that implementation of validated pain assessment tools can have a positive impact on ICU nurses' practice as evidenced by more frequent documented reports of pain assessments. Moreover, evidence showing alterations in use of analgesics and sedatives may suggest that use of pain assessment tools can guide ICU nurses in making more efficient decisions regarding pain and sedation management. Despite heterogeneity in outcome variables and measurements, as well as methodological limitations, this review suggests that implementation of systematic pain assessment may also have a favorable impact on the intensity of pain experienced by critically ill individuals. Most of the reviewed studies that evaluated the impact of pain assessment on the level of pain seem to demonstrate lower, albeit not always statistically significantly, pain ratings after the implementation of a pain assessment tool. However, despite reports of improved outcomes, the effect of systematic pain assessment on duration of MV, adverse events, patient satisfaction, hospital LOS, and mortality is obscured by the small number of studies addressing these outcomes, as well as limitations in statistical power.

The lack of strong evidence to suggest an association between systematic pain assessment and ICU length of stay, mortality, and duration of mechanical ventilation may be due to the fact that, in most of the studies, pain assessment findings were not directly linked to prescribed pharmacological interventions. Indeed, it is acknowledged that use of sedation/analgesia protocols rather than choice of a specific sedation/analgesia scale is associated with outcomes [36]. It is noteworthy that in one of the studies which demonstrated a significant association with the duration of mechanical ventilation physicians had received education regarding analgesics and psychoactive drugs although no analgesia protocol was implemented [15]. Therefore, systematic pain assessment may be a valuable approach to rational and effective analgesia and sedation provided that clinicians possess adequate knowledge regarding pain and sedation management.

Although unrelieved pain in the ICU has been linked to adverse effects, such as prolonged mechanical ventilation, infections, hemodynamic instability, delirium, and depressed immunity [1–3], such complications were rarely addressed in clinical ICU research. Only 3 studies reported on these outcomes with two studies demonstrating statistically significant results in favor of the intervention group. Additionally, evidence with regard to incidence of other complications, such as gastrointestinal bleeding and thromboembolism, is so far inconclusive, probably partially owing to small sample sizes and small rates of such complications. Clearly more research is needed to clarify the effect of systematic pain assessments on ICU complications.

Finally, although patients' satisfaction with their pain management is identified as an important criterion for assessing quality of care [37, 38], this was rarely addressed as an outcome measure within the studies included in this review. Both studies which assessed the impact of pain assessment on patient satisfaction demonstrated high levels of satisfaction either pre- or postimplementation despite patients' reports on the existence of pain. This controversy has been extensively documented in previous studies [39, 40] and it could be attributed to the fact that patients were still receiving care in the hospital while interviewed and they were perhaps reluctant to express dissatisfaction with their care providers [13].

Although practicality of application of behavioral pain tools is an obvious issue of interest, only two groups have assessed feasibility of measurements to find it high, after short only training. This is in line with a feasibility study regarding CPOT, in which the majority of participants (70%) reported that CPOT was helpful and recommended its routine use [41]. It is noteworthy that although behavioral pain tools were not designed specifically for neurosurgical patients, NVPS was reported as easy to use and helpful in a trauma/neurosurgical population in one of the identified studies [13]. Recent advances in application of behavioral pain scales address traumatic brain injury patients [42]. However, more research may be needed to establish the feasibility and validity of behavioral pain tools in this sensitive patient population.

*Limitations.* Limitations of this systematic review stem (a) from the literature search strategy which excluded conference proceedings and (b) from limitations inherent in the identified studies. Specifically, the lack of studies employing randomized controlled approaches highlights the limited level of current evidence with regard to the impact of pain assessment on critically ill patients' outcomes. It is noteworthy that the majority of studies employed pretest-posttest designs, which yield less reliable forms of evidence than concurrent or randomized designs. They are also subject to various types of biases especially in critical care, where facilities, staffing, and patient case-mix are subject to substantial change over time.

In light of the aspect of variation in patient characteristics and in pain assessment tools used between studies, findings should be comparable only in those cases where the same tool was used and only among patients with the same characteristics. Variation in methods of pain assessment across studies and the scarcity of reporting details on who was involved in pain assessment may have confounded the findings of this review further. In some studies pain assessment was conducted by the nursing staff and in other studies by independent assessors with no detailed explanation of their background. This may have accounted in part for the variability of results. Another limitation stems from differences in outcome measures and intervention groups, which precluded use of quantitative syntheses.

## 7. Conclusions and Implications

This systematic review on the impact of systematic pain assessment on critically ill patients' outcomes provided evidence emphasizing the link between systematic pain assessment and short-term critically ill patients' outcomes. Nevertheless, the current level of evidence is insufficient and more high quality randomized clinical studies are essential. In summary it is concluded that (a) implementation of validated pain assessment tools can have a positive impact on the intensity of pain experienced by critically ill individuals, on more efficient use of analgesics and/or sedatives and on ICU clinical practice associated with more frequent assessment and documentation of pain, (b) more study is needed to evaluate the effect of systematic pain assessment on prolonged mechanical ventilation, infections, hemodynamic instability, delirium, depressed immunity, and survival, and (c) the link between systematic pain assessment in critical care and patient satisfaction need to be explored further.

In terms of nursing practice, pain assessment tools should be incorporated into daily practice as it is recommended in the most recent guidelines and quality improvement initiatives [27]. This will assist health care professionals in the prompt identification, early and efficient management of pain and also optimum use of sedatives and analgesics in the ICU. Further, clinical and theoretical training on pain assessment should be included in nursing and medical school curricula, as well as in continuous education in order to help clinicians to better understand the importance of prompt identification and management of pain.

This review highlights the need for further high quality randomized clinical trials to establish the relationship between systematic pain assessment and clinical outcomes in different ICU patients populations. Outcome measures have to be expanded to include long term outcomes since current research seems to demonstrate an association between acute pain management with a decreasing number of long term complications such as chronic pain [43] and posttraumatic stress disorder [44], as well as development of the post-ICU syndrome [45]. As such, it would be crucial to examine how systematic pain assessment would affect those long term outcomes.

## Figures and Tables

**Table 1:** Summary of study characteristics and main outcomes (APACHE: acute physiology and chronic health evaluation; BPS: behavioral pain scale; CPOT: critical pain observation tool; HCP: health care professionals; ICU: intensive care unit; IQR: interquartile range; LOS: length of stay; MV: mechanical ventilation; NA: not assessed; NRS: numerical rating scale; NS: nonsignificant; pts: patients; RASS: Richmond Agitation sedation Scale; SOFA: sequential organ failure assessment).

Authors, year (country)	Study design/aim	Study setting (ICU type) Sample size (*N*)	Type of scale/instrument used and specifics of nurses' HCP's training on use of scales Severity of illness measures	Outcome: level of pain/agitation and documentation of pain assessments	Outcome: use of analgesics and sedatives	Outcome: ICU length of stay (LOS) in days (mean, SD)	Outcome: adverse events/complications	Outcome: hospital length of stay in days (mean, SD)	Outcome: duration of mechanical ventilation in days (mean, SD)	Outcome: days off ventilator	Outcome: patient satisfaction	Outcome: mortality rate
Rose et al., 2013 [9] (Canada)	Preexperimental 2-group pretest-posttest design (preimplementation phase: 5 months, postimplementation: 5 months) Aim: to compare preimplementation to postimplementation practice and outcomes	2 closed ICUs Mixed ICU (medical/surgical/trauma) + cardiovascular ICU *N* = 373 (*n*1 = 189 before implementation; *n*2 = 184 after implementation)	CPOT All nurses received special training (video demonstration and instruction on use of CPOT) Severity of illness measures: SOFA median (IQR) 6 (3–8) for mixed ICU pts before implementation and 4 (3–6) after implementation 8 (5–11) for cardiovascular ICU pts before implementation 8 (6–11) for Cardiovascular ICU pts after implementation	Proportion of pain assessment intervals with pain assessment documented increased from 15% to 64% (*P* < 0.001) (cardiovascular unit); from 22% to 80% (*P* < 0.02) (mixed ICU)	*Median total dose of opioid analgesics* decreased from 5 mg to 4 mg (*P* = 0.02) (cardiovascular ICU) and increased from 27 mg to 75 mg to mixed ICU *Median total dose of benzodiazepines* decreased from 12 mg to 2 mg (*P* < 0.001) (cardiovascular unit); remained unchanged (mixed ICU)	Median LOS ICU in Cardiovascular ICU decreased from 2.0 (IQR, 1.0–5.0) days to 1.8 (IQR, 1.0–3.0) days (*P* = 0.007); in mixed ICU (median, 5.9; IQR, 2.9–13.6 days before and median, 7.0; IQR, 5.0–14.7 days after)	NA	NA	In the cardiovascular ICU median was 0.6 (IQR, 0.3–0.8) days before CPOT and 0.5 (IQR, 0.3–0.8) days after; in the mixed ICU median was 3.9 (IQR, 1.4–10.7) days before implementation and 5.9 (IQR, 3.0–9.1) days after	NA	NA	NA

Radtke et al., 2012 [10] (Germany)	Preexperimental 3-group pretest-posttest design (preimplementation phase (retrospective data collection): 2 months, postimplementation (prospective): 2 months) with 1-year follow-up Aim: to compare the effectiveness of two training strategies standard training for pain assessment sedation and agitation versus modified extended training	3 surgical ICUs *N* = 619 (*n*1 = 241 before implementation; *n*2 = 228 after implementation; *n*3 = 150 follow-up)	NRS, BPS, RASS Standard training for nurses and physicians included lectures, movie, hand-outs one to one instruction Extended modified training including establishment of local support team Severity of illness measures: APACHE II, SOFA	Frequency of monitoring per patient and day for pain (NRS, BPS) ICU 1: pretest 2.3 (1.4–3.2); posttest 4.0 (3.0–5.6); *P* < 0.01 follow-up 4.6 (3.2–5.8); *P* < 0.01 ICU 2: pretest 1.8 (1.3–2.2); post-test: 2.2 (1.8–2.6); p2.6 (1.5–3.5) Follow-up: 2.0 (1.7–3.0); *P* = 0.02 ICU 3: Pretest: (0.0–0.0); post-test: (0.0–0.0); *P* = ns Follow-up: 2.6 (1.5–3.5); *P* < 0.01	N/A Administration of analgesics decreased from 3.87 (SD = 2.59); to 2.47 (SD = 2.56)	ICU 1: pretest 18 (32); posttest 15 (20); follow-up 14 (21); *P* = 0.40 ICU 2: pretest 8 (7); posttest 7 (7); follow-up 4 (3); *P* < 0.01 ICU 3: pretest 9 (8); posttest 7 (8); follow-up 9 (11); *P* = 0.31	N/A Number of complications mean decreased from 4.53 (SD = 5.19) to 1.87 (SD = 1.69) *P* ≤ 0.05	N/A	ICU 1: pretest 355 (697); posttest 281 (448); follow-up 265 (495) *P* = 0.51 ICU 2: pretest 7 (12); posttest 5 (8); follow-up 4 (9); *P* = 0.06 ICU 3: pretest 117 (196); posttest 83 (154); follow-up 149 (244) *P* = 0.74	N/A	N/A	On ICU 2, the measurement of NRS/BPS was associate with reduced mortality (odds ratio (pain) = 0.365 [95% CI: 0.147–0.866], *P* = 0.022

Arbour et al. 2011 [11] (Canada)	PILOT preexperimental 2-group pretest- posttest design (preimplement trauma ICU *n* = 30 medical files: 15 before implementation and 15 after implementation of the CPOT implementation phase: 1 year, postimplementation: 6 months) Aim: to explore the impact of the implementation of the CPOT on pain management and clinical outcomes in mechanically ventilated trauma intensive care unit patients	Trauma ICU *n* = 30 medical Files: 15 before implementation and 15 after implementation of the CPOT	CPOT Nurses were taught how to use the CPOT through a training session and practiced its scoring method with patients' videotapes Severity of illness measures: APACHE II	*Frequency of pain assessments* mean increased from 4.33 (SD = 2.32) to 12.33 (SD = 4.69) *P* ≤ 0.001 No of pain episodes mean increased from 1.13 (SD = 1.13); post implementation 4.27 (SD = 2.09) *P* ≤ 0.001	Morphine equianalgesic dosage mean decreased from 15.60 (SD = 11.35); to 9.37 (SD = 8.94) *P* ≤ 0.10 Administration of sedatives mean decreased from 1.33 (SD = 2.66) to 0.60 (SD = 0.83)	Decreased from 10.53 (SD = 11.16) to 5.33 (SD = 5.86) *P* ≤ 0.10		N/A	(Days) mean decreased from 6.93 (SD = 7.37) to 4.00 (SD = 5.0)	N/A	N/A	N/A

Gélinas et al. 2011 [12] (Canada)	Preexperimental 2-group pretest- posttest design (preimplementation phase: ×3 months, implementation phase: ×3 months post-implementation: ×3 months and 12 months) Aim: to evaluate the implementation of the CPOT on pain assessment and management nursing practices with nonverbal critically ill patients	ICU *N*1 = 60 ICU nurses *N*2 = 90 medical files (30 before implementation, 30 3 months after implementation, 30 12 months after implementation	CPOT All nurses were trained in the use of CPOT Severity of illness measures: NR	*Reports of pain assessments* increased from 3,00 (median) before implementation to 10.50 at 3 months and 12,00 at 12 months after implementation *Documentation of behaviors indicative of the presence of pain* increased from 0,00 (median) before implementation to 1,00 at 3 months and 2,00 at 12 months after implementation. (*U* = 264.00, *P* = 0.005) *Reports of pain reassessments postintervention* frequency (%) increased from 14 (9.92%) before implementation to 28 (43.1%), 3 months and 26 (59.1%), 12 months after implementation	*Number of analgesic bolus administered* (median) decreased from 3,00 before implementation to 1,00 at 3 months and 0,5 at 12 months after implementation (*H* = 11.82, *P* < 0.001) *Total of equianalgesic doses* (median) was similar in the preimplementation group (1,50) and in the 3 months after implementation group (2.25) but was smaller (0,25) at 12 month after implementation *Sedatives*: significant decrease in the number of propofol bolus (*H* = 10.06, *P* < 0.05) and dose (mg) (*H* = 10.29, *P* < 0.05) administered postimplementation	N/A	N/A	N/A	N/A	N/A	N/A	N/A

Topolovec-Vranic et al. 2010 [13] (Canada)	Preexperimental retrospective 2-group pretest-posttest design preimplementation phase: ×4 weeks, postimplement ×4 weeks Aim: to evaluate the effect of the NVPS implementation	Trauma and neurosurgical ICU *n* = 40 medical charts of noncommunicative patients (20 preimplementation and 20 after implementation) and *n* = 26 medical charts of communicative patients (13 before implementation and 13 after implementation) For the patients satisfaction survey *n* = 64 patients (25 before and 39 after the implementation of NVPS)	NVPS patient satisfaction survey (modified of the APS-POQ) Nurses received in-service training for 2 weeks (small group presentations, Pocket education cards) Severity of illness measures: NR	For noncommunicative patients total number of documented pain assessments increased from 457 to 584 after the implementation; the proportion of numerical assessments increased from 131 [29%] to 297 [51%]; *P* < 0.001 and the number of assessments per patient per ICU day increased from 2.2 to 3.4; *P* = 0.02 For communicative patients total number of documented pain assessments increased from 90 to 120; the numerical assessments increased from 8 (9%) to 61 (51%); *P* < 0.001 A trend toward an increase in the number of assessments per patient ICU day was noted (before 6.0, after 10.3; *P* = 0.07) Patients' ratings of their “pain right now” on a scale from 0 to 10 did not differ from before (4.3) to after (3.9) implementation patients reports for the worst pain you had during your ICU stay were lower after implementation (7.2) than before implementation (8.5); *P* = 0.04 The proportion of patients reported the intensity of their pain during their ICU stay as severe was reduced from 55% to 35%	No differences were found in the type of opioid analgesics administered or the amount of medication (morphine equivalents) given per patient or per patient per ICU day when compared from before to after implementation	For noncommunicative patients, days in ICU (standard error of the mean) decreased from 11.9 (2.1) to 10.7 (1.7); *P* = 0.68 after implementation For Communicative patients from 1.0 (0.15) to 1.1 (0.14); *P* = 0.72 after implementation	N/A	N/A	N/A	N/A	High levels of satisfaction with no significant difference between the groups	N/A

Payen et al. 2009 [6] (France)	Concurrent 2-group comparative Both groups followed up until death or ICU discharge or for 30 days in the ICU Aim: to investigate whether an association exists between pain assessments, MV duration, and duration of ICU stay in mechanically ventilated patients receiving analgesia on day 2 of their ICU stay	43 ICUs multicentre *n* = 1144 Mechanically ventilated patients who received analgesia on day 2; *n* = 631 pts not assessed for pain versus *n* = 513 patients assessed for pain on day 2	BPS, VAS, Harris Scale, NRS, verbal descriptor scale Severity of illness measures: SAPS II	Patients assessed for pain were More likely to be assessed for sedation; 91% versus 30%; *P* < 0.01	The group of patients who were assessed for pain (1) were more likely to receive fentanyl *n* (%) 184 (36) versus 179 (28); *P* < 0.01; more dose of sufentanil median (IQR), *μ*g 7.6 (4.2–10.9) versus 5.1 (2.8–7.6); *P* < 0.01 and Less dose of remifentanil, 98 (64–145) versus 149 (77–214); *P* = 0.02 (2) more patients were treated with nonopioids *P* < 0.01 and were more likely to receive multimodal analgesia; *P* < 0.01, and dedicated procedural pain assessment and treatment *P* < 0.01 Patients who were assessed for pain received fewer hypnotics, *n* (%); 384 (75) versus 544 (86); *P* < 0.01 and lower daily doses of midazolam 295 (57) versus 411 (65); *P* < 0.1 Neuromuscular Blocking agents were reduced in patients with pain assessment 35 (7) versus 83 (13); *P* < 0.01	Patients who were assessed for pain had shorter duration of ICU LOS (13 versus 18 days) *P* ≤ 0.01	*Ventilator-acquired pneumonia*, *n* (%) for those not assessed 117 (24) versus 66 (16) *P* < 0.01 *Thromboembolic events, n *(*%*) for those not assessed 13 (3) versus 10 (2); *P* = 0.82 *Gastroduodenal hemorrhage n *(*%*) for those not assessed 8 (2) versus 4 (1); *P* = 0.39 *Central venous catheter colonization, n *(*%*) for those not assessed 28 (6) versus 19 (5); *P* = 0.45	N/A	Patients who were assessed for pain had shorter duration of MV, 8 versus 11 days; *P* < 0.01	Increased odds of weaning from ventilator odds ratio 1.40; 95% confidence interval 1.00–1.98	N/A	Patients not assessed for pain; *n* = 136 (22%) versus patients assessed for pain; *n* = 95 (19%) Unadjusted Odds Ratio (95% CI); 0.91 (0.58–1.43) *P* = 0.69

Williams et al. 2008 [14] (Australia)	Preexperimental 2-group pretest- posttest design (preimplementation phase: 6 m, postimplementation: 6 m) Aim: To evaluate outcomes before and after introduction of scales for analgesia and sedation	General closed ICU *N* = 769 ventilated patients (369 patients before and 400 patients after implementation)	BPS, RASS Education on the use of scales was provided to all staff Severity of illness measures: APACHE II	N/A	For patients who had complete sedation data, the proportion of patients receiving sedatives with or without analgesics during their admission was greater in the after group (88%) than in the before group (57%); *P* < 0.001	It was similar between the groups: 2.3 days before implementation versus 2.6 days after implementation; no significant result (*P* = 0.18)	Incidence of unplanned extubation decreased after implementation (2 unplanned extubations after implementation versus 7 before implementation, *P* = 0.07)	N/A	It did not change significantly after the scales were introduced (median, 24 versus 28 hours) For patients who received mechanical ventilation for 96 hours or longer (24%), mechanical ventilation lasted longer after implementation of the scales (*P* = 0.03)	N/A	N/A	N/A

Chanques et al. 2006 [15] (France)	Preexperimental prospective 2-group pretest-posttest design (preimplementation phase: 21 weeks, postimplementation: 29 weeks) Aim: to measure the impact of implementation of the systematic evaluation of pain and agitation by nurses	Medical-surgical ICU *N* = 230 ICU pts control group, *n* = 100; intervention group, *n* = 130	BPS, NRS, RASS A period of 4 wks of training for physicians and residents who received an oral education and written support based vigorous Assessment and treatment of pain and/or agitation Nurses were trained individually at the bedside to evaluate pain and agitation levels Severity of illness measures: SAPS II	The median observation rate of systematic evaluation of pain and agitation was higher in the intervention group; 75.0 (62.0–90.0) for pain and 77.1 (70.0–90.8) for agitation than the control group 58.6 (40.0–76.7) for pain and 64.0 (50.0–81.7) for agitation; *P* < 0.001 The incidence of pain and agitation was significantly lower in the intervention group than the control group: 63 versus 42% (*P* < 0.002) for pain and 29 versus 12% (*P* < 0.002) for agitation The incidence of severe pain and severe agitation were significantly lower in the intervention group: 36 versus 16% (*P* < 0.001) for pain and 18 versus 5% (*P* < 0.002) for agitation Among pain and agitation events, the rate of severe pain events evaluated by an NRS level >6 and the rate of severe agitation events evaluated by a RASS level >2 was significantly lower in the intervention group: 57 of 176 versus 24 of 168 events (*P* < 0.001) and 42 of 82 versus 9 of 31 events (*P* < 0.03), respectively	Among the analgesic and sedative drugs used during the two phases, tramadol was the only drug used significantly more frequently in the intervention group (16 versus 27%, *P* = 0.05) There was no significant difference among drugs used for continuous sedation in the two groups: Midazolam, propofol, or either was used for continuous sedation, respectively, in 87%, 7%, and 6% in control group versus 74%, 16%, and 10% in the intervention group (*P* = 0.18) Fentanyl, morphine, or either was used for continuous sedation, respectively, in 89%, 2%, and 9% in control group versus 80%, 8%, and 12% in the intervention group (*P* = 0.31)	No significant difference in median length of stay in ICU between the two groups 8.5 (4.0–14.7) in the control versus 7.0 (4.0–13.0) in the intervention group; *P* < 0.38	Marked decrease in nosocomial infections rate in the intervention group; 11/130 (8) versus control group 17/100 (17); *P* < 0.05	N/A	Marked decrease in the total duration of ventilation, hrs; 120 (48–312) in the intervention group versus 65 (24–192) in the control group; *P* = 0.01	N/A	N/A	No significant difference in mortality in ICU between the two groups 12 (12) in the control versus 19 (15) in the intervention group; *P* < 0.76

Voigt et al. 1995 [16] (USA)	Preexperimental 2-group pretest- posttest design Pre- and postimplementation phase duration not reported Aim: to examine the impact of nurses' use of a standardized pain flow sheet to document pain assessments and pharmacologic management on patient -reported pain intensity	Surgical Heart Unit *N* = 61 (*n* = 30 preimplementation group and *n* = 31 postimplementation group)	Standardized pain flow sheet NRS Severity of illness measures: NR	The distribution of pain intensity scores in intervention group was shifted to lower values relative to those in control group for Average pain (*U* = 1150, *P* = 0.001), now pain (*U* = 1094, *P* = 0.015) and least pain (*U* = 1069, *P* = 0.02) Documentation of pain was greatly improved in intervention group for the operative day (*U* = 756, *P* = 0.004) for day 1 (*U* = 1343, *P* < 0.01) and for day 2 (*U* = 627, *P* < 0.01)	The intervention group received more pain medications, morphine equivalents-on day 1 than the control group (*t* = −2.44, *P* = 0.02) No statistical difference was found on the operative day (*t* = 0.37, *P* = 0.72) day 2 (*t* = 0.96, *P* = 0.34) or for the total amount given while in the ICU (*t* = 1.92, *P* = 0.06)	No significant difference between the groups (*U* = 861, *P* = 0.27)	N/A	N/A	No significant difference between the groups (*U* = 975, *P* = 0.43)	N/A	N/A	N/A

Meehan et al. 1995 [17] (USA)	Preexperimental 2-group pretest- posttest design (retrospective phase ×2 months prospective phase from 1992 to 1993) Aim: to examine nursing practice regarding analgesic administration and measure pain intensity and patient satisfaction with pain management practices	Cardiothoracic ICU *N* = 101 (retrospective charts review *n* = 50 and concurrent prospective sample of 51 patients)	VAS, pain relief satisfaction questionnaire Severity of illness measures: NR	The mean VAS score was only available for the prospective group (3.8–4.59)	Amount of time analgesia was held before extubation was decreased in prospective group (5.41 hrs in retrospective group versus 4.25 hrs in prospective) A greater number of patients in the prospective group received procedural analgesia (64%) The prospective group received significantly more analgesia through day 3 than retrospective group (*P* < 0.01) whereas retrospective group received more analgesia later in their ICU stay (*P* = 0.016) In the prospective group more patients received hypnotics (statistics not available)	76% of the prospective group transferred on day 2 or sooner from the ICU versus 54% in the retrospective group	N/A	N/A	No difference in time of extubation between the two groups: (retrospective group mean of 16.11 hrs versus 14.1 hrs in the prospective group)	N/A	96% of the prospective group experienced effective pain management No data were available for the retrospective group	N/A

**Table 2:** Quality Assessment of included studies, EPHPP.

Study	Selection bias	Study design	Confounders	Blinding	Data collection method	Withdrawals and dropouts	Global rating
Rose et al. 2013 [9]	Moderate	Moderate	Strong	Moderate	Strong	Weak	Moderate
Radtke et al. 2012 [10]	Moderate	Moderate	Strong	Moderate	Strong	Weak	Moderate
Arbour et al. 2011 [11]	Moderate	Moderate	Strong	Moderate	Strong	Moderate	Strong
Gélinas et al. 2011 [12]	Moderate	Moderate	Strong	Moderate	Strong	Moderate	Strong
Topolovec-Vranic et al. 2010 [13]	Moderate	Moderate	Strong	Weak	Weak	Strong	Weak
Payen et al. 2009 [6]	Moderate	Moderate	Strong	Moderate	Strong	Moderate	Strong
Williams et al. 2008 [14]	Moderate	Moderate	Moderate	Moderate	Strong	Strong	Strong
Chanques et al. 2006 [15]	Moderate	Moderate	Strong	Moderate	Strong	Moderate	Strong
Voigt et al. 1995 [16]	Weak	Moderate	Weak	Weak	Strong	Strong	Weak
Meehan et al. 1995 [17]	Weak	Strong	Strong	Weak	Strong	Moderate	Weak
